# Supplementary material for: Measuring activity engagement in old age: An exploratory factor analysis
Source: PLoS One. 2021 Dec 6;16(12):e0260996. doi: 10.1371/journal.pone.0260996 (PMC8648112; doi:10.1371/journal.pone.0260996)
Supplement: S1 Appendix — (DOCX) [file pone.0260996.s001.docx]

**S1 Appendix**

**Summary of Multiple Imputation Process**

Seventeen participants did not return the VLS-ALQ; a further 23 returned the questionnaire with missing responses and did not respond to requests to complete these (18 participants missed one item, four participants missed two items and one participant missed five items). The total missing data rate for the VLS-ALQ was 5.2%. All of these missing data were imputed with Multiple Imputation (MI).

Data were imputed as part of a larger analysis examining the relationship between personality, activity engagement, health-related behaviour and cognitive ability using the current sample. Aside from the VLS-ALQ, other measures of interest were age, gender, years of education, deprivation, self-rated health, Mini-Mental State Examination (MMSE) score, trait scores from the NEO Personality Inventory 3 (UK Edition) [1], four composite measures from the 4^th^ (UK) edition of the Wechsler Adult Intelligence Scale (Verbal Comprehension, Perceptual Reasoning, Working Memory and Processing Speed) [2], two composite measures from the 4^th^ (UK) edition of the Wechsler Memory Scale (Older Adult Battery; Auditory Memory and Visual Memory) [3], alcohol consumption (total units per week), diet (number of unhealthy foods typically consumed), smoking pack years (average number of cigarettes smoked per day multiplied by years as a smoker then divided by 20) and physical activity (total MET-minutes per week from the short version of the International Physical Activity Questionnaire). Across all variables, 2.4% of the data was missing.

MI by chained equations was carried out using the mice package in R (version 3.9.0) [4]. Predictive mean matching (PMM) was the selected imputation method for all variables. Based on White et al.’s [5] rule of thumb (the number of imputations should at least be equal to the percentage of missing data), five datasets were imputed, each over 20 iterations. The imputation model included all variables of interest described above.

For the multi-item questionnaires (VLS-ALQ and NEO), MI was conducted on the item responses rather than domain-level scores (i.e., trait scores on the NEO). Evidence suggests that applying MI using PMM to questionnaire data at the item level results in less biased estimates [6].

**References**

1. McCrae RR, Costa PT, Lord W. NEO Personality Inventory - 3 (UK Edition) Manual. Oxford: Hogrefe Ltd.; 2015.

2. Wechsler D. Wechsler Adult Intelligence Scale - Fourth UK Edition (WAIS-IV UK). London: Pearson; 2010.

3. Wechsler D. Wechsler Memory Scale - Fourth UK Edition (WMS-IV UK). London: Pearson; 2010.

4. van Buuren S, Groothuis-Oudshoorn K. mice: Multivariate Imputation by Chained Equations in R. Journal of Statistical Software. 2011;45(3):1-68.

5. White IR, Royston P, Wood AM. Multiple imputation using chained equations: Issues and guidance for practice. Statistics in Medicine. 2011 2011/02/20;30(4):377-99.

6. Eekhout I, de Vet HCW, Twisk JWR, Brand JPL, de Boer MR, Heymans MW. Missing data in a multi-item instrument were best handled by multiple imputation at the item score level. Journal of Clinical Epidemiology. 2014 2014/03/01/;67(3):335-42.
