# Supplementary material for: Measuring activity engagement in old age: An exploratory factor analysis
Source: PLoS One. 2021 Dec 6;16(12):e0260996. doi: 10.1371/journal.pone.0260996 (PMC8648112; doi:10.1371/journal.pone.0260996)
Supplement: S2 Appendix — (DOCX) [file pone.0260996.s002.docx]

**S2 Appendix**

**Items Excluded Prior to Exploratory Factor Analysis.**

| Item | Reason(s) for exclusion |
| --- | --- |
| Woodwork/carpentry | Highly skewed (skew values ranged from 2.27 to 2.51 across five imputed datasets) |
| Collect stamps etc. | Highly skewed (skew values ranged from 2.70 to 3.02 across five imputed datasets) |
| Read for leisure | Highly skewed (skew values ranged from  -2.17 to -2.34 across five imputed datasets) |
| Watch TV news | Highly skewed (skew values ranged from  -2.98 to -3.39 across five imputed datasets);  High kurtosis (kurtosis values ranged from 8.15 to 11.25 across five imputed datasets) |
| Watch TV documentary | Highly skewed (skew values ranged from  -2.06 to -2.19 across five imputed datasets) |
| Use computer | Highly skewed (skew values ranged from  -3.32 to -3.96 across five imputed datasets);  High kurtosis (kurtosis values ranged from 9.86 to 15.03 across five imputed datasets) |
| On-the-job training | Highly skewed (skew values ranged from 2.67 to 2.91 across five imputed datasets) |
| Course at college/university | Highly skewed (skew values ranged from 2.09 to 2.37 across five imputed datasets) |
| Play musical instrument | Did not correlate highly with other items |
| Photography | Did not correlate highly with other items |
| Sewing/knitting/needlework | Did not correlate highly with other items |
| Gardening | Did not correlate highly with other items |
| Exercise (jogging, swimming etc.) | Did not correlate highly with other items |
| Outdoor activities (sailing, fishing etc.) | Did not correlate highly with other items |
| Crossword puzzles etc. | Did not correlate highly with other items |
| Jigsaw puzzles | Did not correlate highly with other items |
| Read newspapers | Did not correlate highly with other items |
| Go to library | Did not correlate highly with other items |
| Listen to radio^a^ | Did not correlate highly with other items |
| Write letters | Did not correlate highly with other items |
| Use electronic calculator | Did not correlate highly with other items |
| Prepare taxes/finances | Did not correlate highly with other items |
| Do mathematical calculations | Did not correlate highly with other items |
| Attend films | Did not correlate highly with other items |
| Attend concert/play^a^ | Did not correlate highly with other items |
| Eat at restaurant | Did not correlate highly with other items |
| Go to pubs/social clubs^b^ | Did not correlate highly with other items |
| Volunteer work | Did not correlate highly with other items |
| Business activities not related to career | Did not correlate highly with other items |
| Study foreign language | Did not correlate highly with other items |
| Travel in foreign country | Did not correlate highly with other items |
| Watch TV comedy/adventure | KMO < .5 |
| Watch TV game shows | Did not correlate highly with other items^c^ |

*Note*. KMO = Kaiser-Meyer-Olkin measure of sampling adequacy. Abbreviated versions of the VLS-ALQ items are included with permission to support the analyses; access to the VLS-ALQ and permission to use the scale in full or in part must be obtained from Professor Roger Dixon (rdixon@ualberta.ca).

^a^ Item reincluded from original 70-item VLS-ALQ. ^b^ New item. ^c^ Item no longer had any correlations > .3 after ‘Watch TV comedy/adventure' was removed.
