# Supplementary material for: Measuring activity engagement in old age: An exploratory factor analysis
Source: PLoS One. 2021 Dec 6;16(12):e0260996. doi: 10.1371/journal.pone.0260996 (PMC8648112; doi:10.1371/journal.pone.0260996)
Supplement: S3 Appendix — (DOCX) [file pone.0260996.s003.docx]

**S3 Appendix**

**Summary Statistics for 29 Items Included in Exploratory Factor Analysis.**

| Item | Mean | SD | KMO |
| --- | --- | --- | --- |
| Household repairs | 2.37 | 2.06 | 0.70 |
| Repair mechanical device | 1.17 | 1.69 | 0.72 |
| Purchase new item requiring set-up | 1.75 | 1.33 | 0.72 |
| Creative writing | 1.38 | 2.18 | 0.68 |
| Recreational sports (tennis, golf etc.) | 1.81 | 2.77 | 0.61 |
| Aerobics (cardiovascular, fitness training etc.) | 2.35 | 2.92 | 0.71 |
| Flexibility training (stretching, yoga etc.) | 3.24 | 3.19 | 0.72 |
| Weight lifting, strength training etc. | 1.28 | 2.47 | 0.62 |
| Card games | 1.95 | 2.65 | 0.73 |
| Board games | 1.35 | 1.83 | 0.74 |
| Knowledge games | 1.45 | 1.73 | 0.69 |
| Word games | 2.08 | 2.24 | 0.67 |
| Read for career/education | 2.13 | 3.09 | 0.79 |
| Go to galleries/museums^b^ | 3.33 | 1.42 | 0.71 |
| Attend public talk | 2.48 | 1.70 | 0.79 |
| Attend sports events^a^ | 1.09 | 1.75 | 0.59 |
| Talk on phone to friends/relatives | 6.32 | 1.61 | 0.62 |
| Visit relatives/friends | 5.39 | 1.53 | 0.58 |
| Go out with friends | 4.99 | 1.68 | 0.70 |
| Attend parties | 2.73 | 1.23 | 0.76 |
| Give dinner/party | 2.51 | 1.59 | 0.71 |
| Attend religious services | 2.09 | 2.43 | 0.58 |
| Engage in prayer/meditation | 2.99 | 3.13 | 0.66 |
| Attend club meetings | 2.71 | 2.53 | 0.76 |
| Attend organised social events | 2.15 | 2.18 | 0.69 |
| Engage in political activities | 1.22 | 1.93 | 0.70 |
| Give public talk | 0.78 | 1.44 | 0.71 |
| Travel outside region | 3.41 | 1.26 | 0.55 |
| Travel outside town | 4.62 | 1.49 | 0.58 |

*Note.* Responses range from 0-8; KMO = Kaiser-Meyer-Olkin measure of sampling adequacy. Abbreviated versions of the VLS-ALQ items are included with permission to support the analyses; access to the VLS-ALQ and permission to use the scale in full or in part must be obtained from Professor Roger Dixon (rdixon@ualberta.ca).

^a^ Item reincluded from original 70-item VLS-ALQ. ^b^ New item.
