# Supplementary material for: Measuring activity engagement in old age: An exploratory factor analysis
Source: PLoS One. 2021 Dec 6;16(12):e0260996. doi: 10.1371/journal.pone.0260996 (PMC8648112; doi:10.1371/journal.pone.0260996)
Supplement: S4 Appendix — (DOCX) [file pone.0260996.s004.docx]

**S4 Appendix**

**Parallel Analysis Scree Plot**


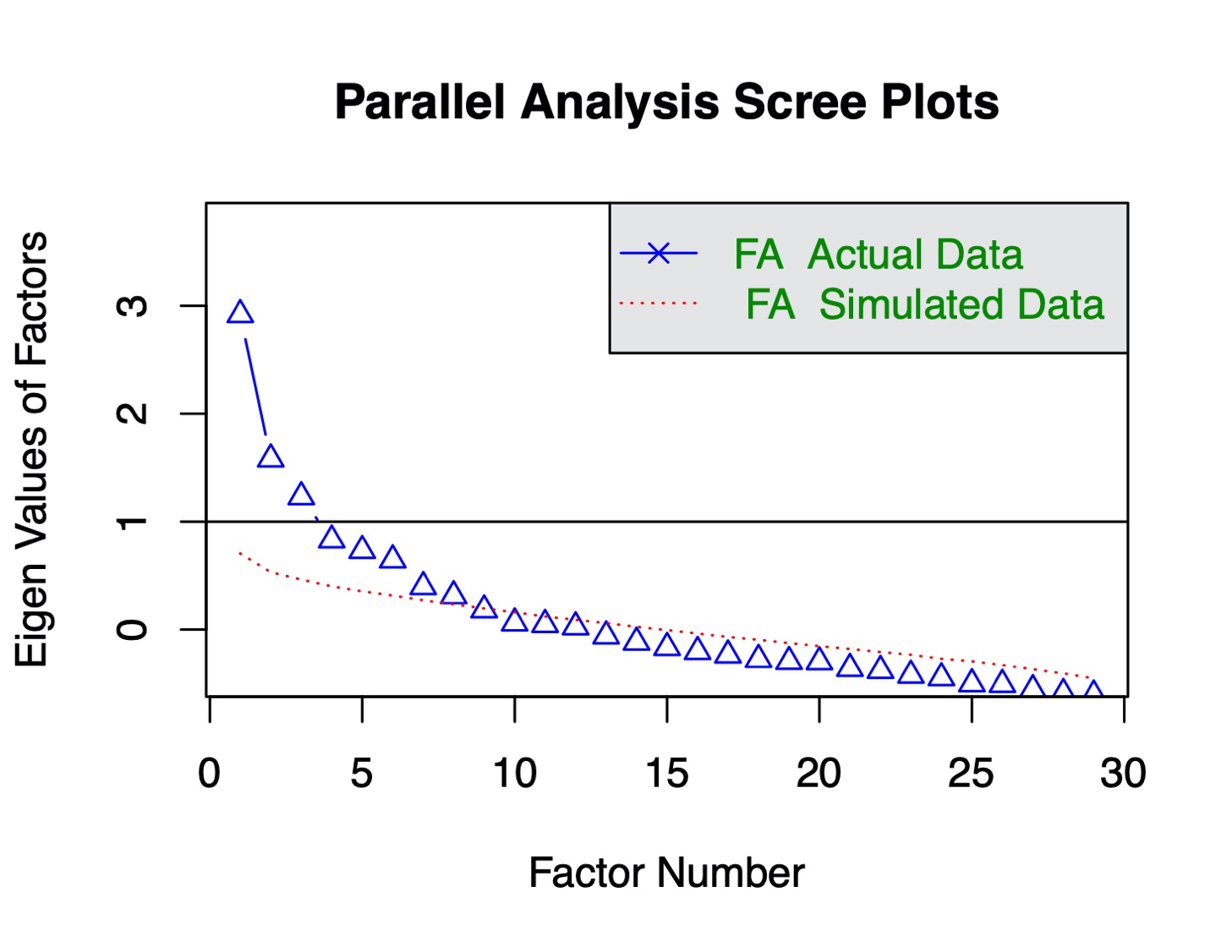


*Note.* Parallel analysis compares the eigenvalues of the observed data (blue triangles) to those obtained from simulated random data of the same size (red dashed line) – only the observed eigenvalues that are greater than those in the random data are retained.
