# Supplementary material for: Measuring activity engagement in old age: An exploratory factor analysis
Source: PLoS One. 2021 Dec 6;16(12):e0260996. doi: 10.1371/journal.pone.0260996 (PMC8648112; doi:10.1371/journal.pone.0260996)
Supplement: S5 Appendix — (DOCX) [file pone.0260996.s005.docx]

**S5 Appendix**

**Six-Factor Model Structure Matrix**

|  | Factor | | | | | |
| --- | --- | --- | --- | --- | --- | --- |
| Item | 1. Man | 2. Int | 3. Gam | 4. Rel | 5. Exe | 6. Soc |
| Do household repairs (for example, painting or leaky faucets) | **0.86** | 0.15 | 0.08 | -0.03 | 0.14 | -0.06 |
| Repair a mechanical device (for example, a car or lawn mower) | **0.73** | 0.22 | 0.08 | -0.01 | 0.13 | -0.05 |
| Purchase a new item requiring some set-up or assembly | **0.62** | 0.11 | 0.12 | -0.01 | 0.09 | -0.11 |
| Engage in creative writing, writing poems, writing newspaper articles, etc. | 0.22 | **0.57** | 0.06 | 0.14 | 0.08 | 0.05 |
| Read books or magazines as part of my job, career, or formal education | 0.13 | **0.53** | 0.13 | 0.00 | 0.14 | 0.05 |
| Go to galleries or museums | -0.02 | **0.46** | 0.21 | 0.06 | 0.21 | 0.16 |
| Attend a public lecture or talk | 0.01 | **0.57** | 0.21 | 0.20 | 0.21 | 0.06 |
| Engage in political activities (for example, neighbourhood organisation) | 0.17 | **0.47** | 0.10 | 0.06 | 0.16 | 0.02 |
| Give a public talk or lecture (for example, to a club, service organisation, etc.) | 0.15 | **0.50** | 0.01 | 0.14 | 0.13 | -0.05 |
| Do aerobics (for example, cardiovascular, fitness training, or workout) | 0.14 | 0.18 | 0.11 | 0.05 | **0.65** | -0.01 |
| Do flexibility training (for example, stretching, yoga, or tai chi) | 0.01 | 0.21 | 0.18 | 0.07 | **0.51** | 0.06 |
| Do weight lifting, strength training, or calisthenics | 0.14 | 0.17 | 0.02 | -0.06 | **0.76** | 0.00 |
| Play card games (for example, Bridge) | 0.02 | 0.06 | **0.46** | 0.08 | 0.10 | 0.07 |
| Play board games (for example, chess or checkers) | 0.14 | 0.17 | **0.63** | 0.05 | 0.10 | 0.03 |
| Play knowledge games (for example, Trivial Pursuit) | 0.09 | 0.16 | **0.65** | 0.06 | 0.06 | 0.09 |
| Play word games (for example, Scrabble) | 0.00 | 0.07 | **0.63** | 0.04 | 0.02 | 0.13 |
| Talk on the phone to friends, or relatives | -0.14 | 0.03 | 0.14 | 0.15 | 0.03 | **0.37** |
| Visit relatives, friends, or neighbours | -0.08 | 0.08 | 0.14 | 0.04 | 0.01 | **1.00** |
| Go out with friends | -0.18 | 0.08 | 0.06 | 0.03 | -0.05 | **0.36** |
| Attend church or other religious services | -0.03 | 0.13 | 0.06 | **0.90** | -0.05 | 0.02 |
| Engage in prayer, meditation, or philosophical contemplation | 0.01 | 0.22 | 0.14 | **0.56** | 0.13 | 0.04 |
| Attend organised social events (for example, activities at the community centre or church social groups) | -0.04 | 0.23 | 0.11 | **0.50** | 0.08 | 0.17 |

*Note*. Man = Manual, Int = Intellectual, Gam = Games, Rel = Religious, Exe = Exercise, Soc = Social. **Bold** = coefficient > .3. VLS-ALQ items are included with permission to support the analyses; permission to use the VLS-ALQ in full or in part must be obtained from Professor Roger Dixon (rdixon@ualberta.ca)
