# Supplementary material for: Measuring activity engagement in old age: An exploratory factor analysis
Source: PLoS One. 2021 Dec 6;16(12):e0260996. doi: 10.1371/journal.pone.0260996 (PMC8648112; doi:10.1371/journal.pone.0260996)
Supplement: S6 Appendix — (DOCX) [file pone.0260996.s006.docx]

**S6 Appendix**

**Pattern/Structure Matrices for Alternative Factor Solutions**

**Table A. Three-Factor Model Pattern Matrix**

|  | Factor | | |  |
| --- | --- | --- | --- | --- |
| Item | 1. | 2. | 3. | *h^2^* |
| Household repairs | -0.03 | **0.84** | 0.00 | 0.70 |
| Repair mechanical device | 0.06 | **0.73** | 0.00 | 0.55 |
| Purchase new item requiring set-up | -0.03 | **0.62** | 0.06 | 0.39 |
| Creative writing | **0.49** | 0.18 | -0.08 | 0.29 |
| Card games | 0.01 | -0.02 | **0.46** | 0.21 |
| Board games | 0.06 | 0.09 | **0.60** | 0.40 |
| Knowledge games | 0.02 | 0.03 | **0.65** | 0.43 |
| Word games | -0.05 | -0.05 | **0.66** | 0.41 |
| Read for career/education | **0.37** | 0.11 | 0.02 | 0.17 |
| Go to galleries/museums | **0.41** | -0.06 | 0.11 | 0.20 |
| Attend public talk | **0.61** | -0.05 | 0.06 | 0.38 |
| Attend religious services | **0.37** | -0.10 | -0.01 | 0.13 |
| Engage in prayer/meditation | **0.38** | -0.06 | 0.05 | 0.15 |
| Attend club meetings | **0.50** | -0.09 | 0.05 | 0.26 |
| Attend organised social events | **0.46** | -0.12 | 0.01 | 0.22 |
| Engage in political activities | **0.41** | 0.14 | -0.02 | 0.20 |
| Give public talk | **0.45** | 0.12 | -0.11 | 0.22 |

*Note*. **Bold** = loading > .3; *h^2^* = communality. Abbreviated versions of the VLS-ALQ items are included with permission to support the analyses; access to the VLS-ALQ and permission to use the scale in full or in part must be obtained from Professor Roger Dixon (rdixon@ualberta.ca).

**Table B. Three-Factor Model Structure Matrix**

|  | Factor | | |
| --- | --- | --- | --- |
| Item | 1. | 2. | 3. |
| Household repairs | 0.09 | **0.84** | 0.08 |
| Repair mechanical device | 0.16 | **0.74** | 0.08 |
| Purchase new item requiring set-up | 0.07 | **0.63** | 0.11 |
| Creative writing | **0.50** | 0.24 | 0.05 |
| Card games | 0.11 | 0.02 | **0.46** |
| Board games | 0.21 | 0.15 | **0.62** |
| Knowledge games | 0.17 | 0.10 | **0.65** |
| Word games | 0.09 | 0.01 | **0.64** |
| Read for career/education | **0.39** | 0.17 | 0.11 |
| Go to galleries/museums | **0.43** | 0.00 | 0.20 |
| Attend public talk | **0.61** | 0.04 | 0.19 |
| Attend religious services | **0.35** | -0.05 | 0.06 |
| Engage in prayer/meditation | **0.38** | 0.00 | 0.12 |
| Attend club meetings | **0.50** | -0.02 | 0.15 |
| Attend organised social events | **0.45** | -0.06 | 0.10 |
| Engage in political activities | **0.42** | 0.19 | 0.08 |
| Give public talk | **0.45** | 0.17 | 0.00 |

*Note*. **Bold** = loading > .3. Abbreviated versions of the VLS-ALQ items are included with permission to support the analyses; access to the VLS-ALQ and permission to use the scale in full or in part must be obtained from Professor Roger Dixon (rdixon@ualberta.ca).

**Table C. Four-Factor Model Pattern Matrix**

|  | Factor | | | |  |
| --- | --- | --- | --- | --- | --- |
| Item | 1. | 2. | 3. | 4. | *h^2^* |
| Household repairs | -0.03 | **0.86** | 0.00 | 0.01 | 0.73 |
| Repair mechanical device | 0.06 | **0.72** | 0.00 | 0.00 | 0.54 |
| Purchase new item requiring set-up | -0.02 | **0.62** | 0.06 | 0.00 | 0.39 |
| Creative writing | **0.45** | 0.16 | -0.07 | 0.07 | 0.26 |
| Aerobics (cardiovascular, fitness training etc.) | **0.32** | 0.10 | 0.03 | -0.05 | 0.12 |
| Flexibility training (stretching, yoga etc.) | **0.36** | -0.05 | 0.11 | -0.03 | 0.15 |
| Weight lifting, strength training etc. | **0.36** | 0.09 | -0.04 | -0.15 | 0.15 |
| Card games | -0.01 | -0.02 | **0.46** | 0.04 | 0.22 |
| Board games | 0.06 | 0.08 | **0.61** | -0.01 | 0.41 |
| Knowledge games | 0.01 | 0.03 | **0.64** | 0.00 | 0.42 |
| Word games | -0.05 | -0.05 | **0.65** | -0.01 | 0.41 |
| Read for career/education | **0.47** | 0.07 | 0.01 | -0.11 | 0.22 |
| Go to galleries/museums | **0.49** | -0.10 | 0.10 | -0.06 | 0.25 |
| Attend public talk | **0.60** | -0.08 | 0.05 | 0.07 | 0.39 |
| Attend religious services | -0.06 | 0.01 | -0.01 | **0.86** | 0.73 |
| Engage in prayer/meditation | 0.11 | 0.01 | 0.05 | **0.55** | 0.35 |
| Attend club meetings | **0.42** | -0.08 | 0.06 | 0.17 | 0.25 |
| Attend organised social events | 0.20 | -0.06 | 0.02 | **0.49** | 0.33 |
| Engage in political activities | **0.43** | 0.11 | -0.03 | -0.01 | 0.21 |
| Give public talk | **0.45** | 0.10 | -0.12 | 0.05 | 0.22 |

*Note*. **Bold** = loading > .3; *h^2^* = communality. Abbreviated versions of the VLS-ALQ items are included with permission to support the analyses; access to the VLS-ALQ and permission to use the scale in full or in part must be obtained from Professor Roger Dixon (rdixon@ualberta.ca).

**Table D. Four-Factor Model Structure Matrix**

|  | Factor | | | |
| --- | --- | --- | --- | --- |
| Item | 1. | 2. | 3. | 4. |
| Household repairs | 0.12 | **0.85** | 0.08 | -0.03 |
| Repair mechanical device | 0.18 | **0.73** | 0.08 | -0.01 |
| Purchase new item requiring set-up | 0.10 | **0.62** | 0.11 | -0.02 |
| Creative writing | **0.48** | 0.23 | 0.05 | 0.16 |
| Aerobics (cardiovascular, fitness training etc.) | **0.33** | 0.15 | 0.11 | 0.03 |
| Flexibility training (stretching, yoga etc.) | **0.37** | 0.02 | 0.18 | 0.06 |
| Weight lifting, strength training etc. | **0.34** | 0.15 | 0.04 | -0.08 |
| Card games | 0.11 | 0.03 | **0.46** | 0.08 |
| Board games | 0.21 | 0.15 | **0.63** | 0.06 |
| Knowledge games | 0.17 | 0.10 | **0.65** | 0.06 |
| Word games | 0.09 | 0.01 | **0.63** | 0.04 |
| Read for career/education | **0.46** | 0.15 | 0.11 | 0.00 |
| Go to galleries/museums | **0.48** | -0.01 | 0.20 | 0.06 |
| Attend public talk | **0.61** | 0.02 | 0.19 | 0.21 |
| Attend religious services | 0.14 | -0.03 | 0.05 | **0.85** |
| Engage in prayer/meditation | 0.25 | 0.02 | 0.13 | **0.58** |
| Attend club meetings | **0.46** | -0.02 | 0.16 | 0.27 |
| Attend organised social events | **0.31** | -0.04 | 0.10 | **0.54** |
| Engage in political activities | **0.44** | 0.18 | 0.08 | 0.08 |
| Give public talk | **0.45** | 0.16 | 0.00 | 0.13 |

*Note*. **Bold** = loading > .3. Abbreviated versions of the VLS-ALQ items are included with permission to support the analyses; access to the VLS-ALQ and permission to use the scale in full or in part must be obtained from Professor Roger Dixon (rdixon@ualberta.ca).

**Table E. Five-Factor Model Pattern Matrix**

|  | Factor | | | | |  |
| --- | --- | --- | --- | --- | --- | --- |
| Item | 1. | 2. | 3. | 4. | 5. | *h^2^* |
| Household repairs | **0.86** | -0.02 | 0.00 | 0.00 | 0.02 | 0.73 |
| Repair mechanical device | **0.71** | 0.09 | 0.00 | 0.00 | 0.00 | 0.54 |
| Purchase new item requiring set-up | **0.62** | -0.02 | 0.06 | 0.00 | 0.00 | 0.39 |
| Creative writing | 0.12 | **0.57** | -0.06 | 0.05 | -0.08 | 0.35 |
| Aerobics (cardiovascular, fitness training etc.) | 0.05 | -0.02 | 0.02 | 0.05 | **0.65** | 0.43 |
| Flexibility training (stretching, yoga etc.) | -0.09 | 0.07 | 0.12 | 0.05 | **0.49** | 0.29 |
| Weight lifting, strength training etc. | 0.03 | -0.01 | -0.06 | -0.05 | **0.76** | 0.59 |
| Card games | -0.02 | -0.05 | **0.46** | 0.05 | 0.06 | 0.22 |
| Board games | 0.09 | 0.03 | **0.62** | -0.01 | 0.01 | 0.41 |
| Knowledge games | 0.03 | 0.03 | **0.65** | 0.00 | -0.03 | 0.43 |
| Word games | -0.04 | -0.04 | **0.65** | -0.01 | -0.03 | 0.40 |
| Read for career/education | 0.02 | **0.55** | 0.03 | -0.11 | -0.01 | 0.30 |
| Go to galleries/museums | -0.14 | **0.44** | 0.12 | -0.03 | 0.10 | 0.25 |
| Attend public talk | -0.11 | **0.53** | 0.09 | 0.09 | 0.07 | 0.35 |
| Attend religious services | 0.01 | -0.03 | -0.02 | **0.90** | -0.04 | 0.79 |
| Engage in prayer/meditation | -0.01 | 0.08 | 0.06 | **0.55** | 0.11 | 0.35 |
| Attend organised social events | -0.06 | 0.13 | 0.04 | **0.48** | 0.05 | 0.28 |
| Engage in political activities | 0.08 | **0.46** | 0.00 | -0.01 | 0.02 | 0.23 |
| Give public talk | 0.06 | **0.50** | -0.10 | 0.05 | 0.00 | 0.26 |

*Note*. **Bold** = loading > .3; *h^2^* = communality. Abbreviated versions of the VLS-ALQ items are included with permission to support the analyses; access to the VLS-ALQ and permission to use the scale in full or in part must be obtained from Professor Roger Dixon (rdixon@ualberta.ca).

**Table F. Five-Factor Model Structure Matrix**

|  | Factor | | | | |
| --- | --- | --- | --- | --- | --- |
| Item | 1. | 2. | 3. | 4. | 5. |
| Household repairs | **0.86** | 0.15 | 0.08 | -0.02 | 0.14 |
| Repair mechanical device | **0.73** | 0.23 | 0.08 | -0.01 | 0.13 |
| Purchase new item requiring set-up | **0.62** | 0.11 | 0.11 | -0.02 | 0.09 |
| Creative writing | 0.22 | **0.57** | 0.06 | 0.15 | 0.08 |
| Aerobics (cardiovascular, fitness training etc.) | 0.14 | 0.18 | 0.10 | 0.05 | **0.65** |
| Flexibility training (stretching, yoga etc.) | 0.01 | 0.21 | 0.19 | 0.08 | **0.51** |
| Weight lifting, strength training etc. | 0.14 | 0.18 | 0.02 | -0.06 | **0.76** |
| Card games | 0.02 | 0.06 | **0.46** | 0.09 | 0.10 |
| Board games | 0.15 | 0.17 | **0.63** | 0.05 | 0.11 |
| Knowledge games | 0.09 | 0.16 | **0.65** | 0.06 | 0.06 |
| Word games | 0.01 | 0.08 | **0.63** | 0.04 | 0.02 |
| Read for career/education | 0.14 | **0.54** | 0.13 | 0.00 | 0.14 |
| Go to galleries/museums | -0.02 | **0.46** | 0.21 | 0.06 | 0.21 |
| Attend public talk | 0.01 | **0.57** | 0.21 | 0.20 | 0.21 |
| Attend religious services | -0.03 | 0.13 | 0.06 | **0.89** | -0.05 |
| Engage in prayer/meditation | 0.01 | 0.22 | 0.13 | **0.57** | 0.13 |
| Attend organised social events | -0.04 | 0.23 | 0.11 | **0.51** | 0.08 |
| Engage in political activities | 0.17 | **0.48** | 0.10 | 0.07 | 0.16 |
| Give public talk | 0.15 | **0.50** | 0.01 | 0.13 | 0.13 |

*Note*. **Bold** = loading > .3. Abbreviated versions of the VLS-ALQ items are included with permission to support the analyses; access to the VLS-ALQ and permission to use the scale in full or in part must be obtained from Professor Roger Dixon (rdixon@ualberta.ca).

**Table G. Seven-Factor Model Pattern Matrix**

|  | Factor | | | | | | |  |
| --- | --- | --- | --- | --- | --- | --- | --- | --- |
| Item | 1. | 2. | 3. | 4. | 5. | 6. | 7. | *h^2^* |
| Household repairs | **0.86** | -0.03 | 0.00 | 0.01 | 0.02 | 0.02 | 0.01 | 0.74 |
| Repair mechanical device | **0.71** | 0.08 | 0.01 | 0.00 | 0.01 | 0.02 | -0.05 | 0.54 |
| Purchase new item requiring set-up | **0.60** | -0.01 | 0.06 | 0.00 | -0.01 | -0.07 | 0.11 | 0.41 |
| Creative writing | 0.13 | **0.58** | -0.04 | 0.04 | -0.06 | 0.04 | -0.11 | 0.38 |
| Aerobics (cardiovascular, fitness training etc.) | 0.04 | -0.01 | 0.02 | 0.05 | **0.63** | -0.03 | 0.06 | 0.43 |
| Flexibility training (stretching, yoga etc.) | -0.08 | 0.06 | 0.12 | 0.05 | **0.49** | 0.02 | -0.01 | 0.29 |
| Weight lifting, strength training etc. | 0.03 | -0.01 | -0.06 | -0.06 | **0.77** | 0.00 | 0.00 | 0.60 |
| Card games | -0.02 | -0.05 | **0.46** | 0.05 | 0.05 | 0.00 | 0.05 | 0.22 |
| Board games | 0.08 | 0.02 | **0.65** | -0.01 | 0.02 | -0.04 | -0.07 | 0.43 |
| Knowledge games | 0.02 | 0.03 | **0.64** | -0.01 | -0.04 | 0.00 | 0.08 | 0.43 |
| Word games | -0.04 | -0.04 | **0.63** | -0.01 | -0.04 | 0.05 | 0.01 | 0.39 |
| Read for career/education | 0.01 | **0.56** | 0.02 | -0.10 | -0.01 | 0.00 | 0.11 | 0.32 |
| Go to galleries/museums | -0.12 | **0.41** | 0.12 | -0.03 | 0.11 | 0.11 | -0.02 | 0.25 |
| Attend public talk | -0.11 | **0.52** | 0.10 | 0.09 | 0.08 | -0.01 | 0.02 | 0.34 |
| Talk on phone to friends/relatives | -0.11 | -0.03 | 0.08 | 0.13 | 0.03 | **0.34** | 0.05 | 0.18 |
| Visit relatives/friends | 0.01 | 0.00 | -0.01 | -0.01 | -0.01 | **1.00** | 0.01 | 1.00 |
| Attend religious services | 0.01 | -0.03 | -0.02 | **0.91** | -0.04 | -0.02 | 0.04 | 0.83 |
| Engage in prayer/meditation | 0.01 | 0.07 | 0.08 | **0.54** | 0.13 | 0.01 | -0.15 | 0.37 |
| Attend organised social events | -0.05 | 0.12 | 0.02 | **0.47** | 0.06 | 0.13 | -0.02 | 0.29 |
| Engage in political activities | 0.08 | **0.45** | 0.02 | -0.02 | 0.04 | 0.00 | -0.08 | 0.24 |
| Give public talk | 0.03 | **0.52** | -0.10 | 0.05 | 0.00 | -0.09 | 0.12 | 0.30 |
| Travel outside region | 0.00 | 0.02 | 0.03 | 0.02 | 0.00 | 0.02 | **0.70** | 0.50 |
| Travel outside town | 0.04 | 0.00 | -0.01 | -0.02 | 0.06 | 0.02 | **0.61** | 0.40 |

*Note*. **Bold** = loading > .3; *h^2^* = communality. Abbreviated versions of the VLS-ALQ items are included with permission to support the analyses; access to the VLS-ALQ and permission to use the scale in full or in part must be obtained from Professor Roger Dixon (rdixon@ualberta.ca).

**Table H. Seven-Factor Model Structure Matrix**

|  | Factor | | | | | | |
| --- | --- | --- | --- | --- | --- | --- | --- |
| Item | 1. | 2. | 3. | 4. | 5. | 6. | 7. |
| Household repairs | **0.86** | 0.15 | 0.08 | -0.02 | 0.14 | -0.06 | 0.10 |
| Repair mechanical device | **0.73** | 0.23 | 0.09 | -0.01 | 0.13 | -0.04 | 0.03 |
| Purchase new item requiring set-up | **0.62** | 0.11 | 0.12 | -0.02 | 0.09 | -0.11 | 0.17 |
| Creative writing | 0.22 | **0.59** | 0.07 | 0.14 | 0.08 | 0.06 | -0.09 |
| Aerobics (cardiovascular, fitness training etc.) | 0.14 | 0.17 | 0.10 | 0.05 | **0.65** | -0.01 | 0.14 |
| Flexibility training (stretching, yoga etc.) | 0.01 | 0.20 | 0.19 | 0.07 | **0.51** | 0.06 | 0.05 |
| Weight lifting, strength training etc. | 0.14 | 0.16 | 0.02 | -0.06 | **0.77** | 0.00 | 0.09 |
| Card games | 0.02 | 0.05 | **0.46** | 0.08 | 0.10 | 0.07 | 0.11 |
| Board games | 0.15 | 0.16 | **0.64** | 0.05 | 0.11 | 0.04 | 0.01 |
| Knowledge games | 0.09 | 0.15 | **0.65** | 0.06 | 0.06 | 0.09 | 0.15 |
| Word games | 0.00 | 0.07 | **0.62** | 0.04 | 0.02 | 0.13 | 0.08 |
| Read for career/education | 0.13 | **0.54** | 0.13 | 0.00 | 0.14 | 0.04 | 0.12 |
| Go to galleries/museums | -0.02 | **0.44** | 0.21 | 0.06 | 0.21 | 0.16 | 0.00 |
| Attend public talk | 0.01 | **0.55** | 0.21 | 0.20 | 0.21 | 0.06 | 0.04 |
| Talk on phone to friends/relatives | -0.13 | 0.02 | 0.13 | 0.15 | 0.03 | **0.37** | 0.08 |
| Visit relatives/friends | -0.08 | 0.07 | 0.13 | 0.04 | 0.02 | **1.00** | 0.08 |
| Attend religious services | -0.03 | 0.13 | 0.06 | **0.91** | -0.05 | 0.02 | 0.02 |
| Engage in prayer/meditation | 0.01 | 0.21 | 0.14 | **0.56** | 0.14 | 0.04 | -0.13 |
| Attend organised social events | -0.04 | 0.22 | 0.11 | **0.50** | 0.08 | 0.17 | -0.01 |
| Engage in political activities | 0.17 | **0.47** | 0.11 | 0.06 | 0.16 | 0.02 | -0.06 |
| Give public talk | 0.15 | **0.52** | 0.01 | 0.13 | 0.13 | -0.05 | 0.11 |
| Travel outside region | 0.08 | 0.04 | 0.12 | 0.02 | 0.09 | 0.08 | **0.71** |
| Travel outside town | 0.11 | 0.03 | 0.08 | -0.02 | 0.14 | 0.07 | **0.62** |

*Note*. **Bold** = loading > .3. Abbreviated versions of the VLS-ALQ items are included with permission to support the analyses; access to the VLS-ALQ and permission to use the scale in full or in part must be obtained from Professor Roger Dixon (rdixon@ualberta.ca).

**Table I. Eight-Factor Model Pattern Matrix**

|  | Factor | | | | | | | |  |
| --- | --- | --- | --- | --- | --- | --- | --- | --- | --- |
| Item | 1. | 2. | 3. | 4. | 5. | 6. | 7. | 8. | *h^2^* |
| Household repairs | **0.87** | -0.02 | -0.01 | 0.00 | 0.02 | 0.02 | 0.01 | -0.02 | 0.74 |
| Repair mechanical device | **0.69** | 0.08 | 0.01 | 0.00 | 0.02 | 0.02 | -0.06 | 0.15 | 0.57 |
| Purchase new item requiring set-up | **0.62** | 0.00 | 0.04 | 0.00 | -0.03 | -0.07 | 0.13 | -0.11 | 0.42 |
| Creative writing | 0.14 | **0.58** | -0.05 | 0.04 | -0.07 | 0.03 | -0.10 | -0.05 | 0.38 |
| Aerobics (cardiovascular, fitness training etc.) | 0.01 | -0.01 | 0.02 | 0.05 | **0.67** | -0.03 | 0.06 | 0.10 | 0.47 |
| Flexibility training (stretching, yoga etc.) | -0.04 | 0.07 | 0.09 | 0.04 | **0.49** | 0.02 | 0.00 | -0.28 | 0.37 |
| Weight lifting, strength training etc. | 0.03 | 0.00 | -0.06 | -0.06 | **0.75** | 0.00 | 0.00 | 0.02 | 0.56 |
| Card games | 0.01 | -0.05 | **0.43** | 0.05 | 0.04 | 0.01 | 0.06 | -0.14 | 0.23 |
| Board games | 0.10 | 0.03 | **0.63** | -0.01 | 0.02 | -0.04 | -0.07 | -0.10 | 0.42 |
| Knowledge games | -0.02 | 0.03 | **0.70** | 0.00 | -0.02 | 0.00 | 0.06 | 0.18 | 0.53 |
| Word games | -0.03 | -0.04 | **0.62** | -0.01 | -0.04 | 0.05 | 0.00 | -0.04 | 0.38 |
| Read for career/education | 0.01 | **0.55** | 0.03 | -0.10 | -0.02 | 0.00 | 0.11 | 0.00 | 0.31 |
| Go to galleries/museums | -0.09 | **0.43** | 0.11 | -0.04 | 0.08 | 0.10 | -0.02 | -0.16 | 0.27 |
| Attend public talk | -0.11 | **0.53** | 0.10 | 0.09 | 0.08 | -0.01 | 0.02 | -0.02 | 0.35 |
| Attend sports events | 0.12 | 0.02 | 0.13 | 0.01 | 0.10 | 0.03 | -0.07 | **0.52** | 0.34 |
| Talk on phone to friends/relatives | -0.13 | -0.02 | 0.08 | 0.13 | 0.04 | **0.35** | 0.05 | 0.07 | 0.18 |
| Visit relatives/friends | 0.01 | -0.01 | -0.01 | -0.01 | 0.00 | **1.00** | 0.00 | 0.00 | 1.00 |
| Go out with friends | -0.15 | 0.09 | 0.00 | -0.01 | -0.06 | **0.33** | 0.10 | -0.04 | 0.17 |
| Attend religious services | 0.00 | -0.03 | -0.02 | **0.92** | -0.04 | -0.02 | 0.03 | 0.03 | 0.84 |
| Engage in prayer/meditation | 0.03 | 0.08 | 0.07 | **0.54** | 0.11 | 0.01 | -0.14 | -0.14 | 0.38 |
| Attend organised social events | -0.04 | 0.12 | 0.02 | **0.46** | 0.05 | 0.14 | -0.02 | -0.02 | 0.29 |
| Engage in political activities | 0.09 | **0.44** | 0.02 | -0.02 | 0.05 | 0.00 | -0.09 | 0.00 | 0.24 |
| Give public talk | 0.00 | **0.53** | -0.09 | 0.06 | 0.02 | -0.08 | 0.10 | 0.19 | 0.34 |
| Travel outside region | 0.02 | 0.02 | 0.03 | 0.02 | 0.00 | 0.02 | **0.72** | -0.04 | 0.53 |
| Travel outside town | 0.05 | 0.00 | -0.01 | -0.02 | 0.07 | 0.03 | **0.59** | 0.01 | 0.38 |

*Note*. **Bold** = loading > .3; *h^2^* = communality. Abbreviated versions of the VLS-ALQ items are included with permission to support the analyses; access to the VLS-ALQ and permission to use the scale in full or in part must be obtained from Professor Roger Dixon (rdixon@ualberta.ca).

**Table J. Eight-Factor Model Structure Matrix**

|  | Factor | | | | | | | |
| --- | --- | --- | --- | --- | --- | --- | --- | --- |
| Item | 1. | 2. | 3. | 4. | 5. | 6. | 7. | 8. |
| Household repairs | **0.86** | 0.15 | 0.08 | -0.02 | 0.15 | -0.06 | 0.08 | 0.12 |
| Repair mechanical device | **0.73** | 0.22 | 0.10 | -0.01 | 0.14 | -0.04 | 0.01 | 0.26 |
| Purchase new item requiring set-up | **0.62** | 0.11 | 0.11 | -0.01 | 0.09 | -0.11 | 0.17 | 0.01 |
| Creative writing | 0.22 | **0.59** | 0.06 | 0.14 | 0.08 | 0.06 | -0.09 | -0.02 |
| Aerobics (cardiovascular, fitness training etc.) | 0.14 | 0.17 | 0.11 | 0.05 | **0.67** | -0.01 | 0.13 | 0.08 |
| Flexibility training (stretching, yoga etc.) | 0.01 | 0.20 | 0.17 | 0.07 | **0.52** | 0.07 | 0.06 | -0.30 |
| Weight lifting, strength training etc. | 0.14 | 0.17 | 0.03 | -0.06 | **0.74** | 0.00 | 0.07 | 0.00 |
| Card games | 0.03 | 0.05 | **0.44** | 0.08 | 0.10 | 0.07 | 0.11 | -0.15 |
| Board games | 0.15 | 0.16 | **0.63** | 0.05 | 0.11 | 0.04 | 0.01 | -0.09 |
| Knowledge games | 0.09 | 0.15 | **0.70** | 0.06 | 0.07 | 0.09 | 0.15 | 0.18 |
| Word games | 0.01 | 0.06 | **0.61** | 0.04 | 0.03 | 0.13 | 0.07 | -0.05 |
| Read for career/education | 0.13 | **0.54** | 0.13 | 0.00 | 0.14 | 0.05 | 0.12 | 0.02 |
| Go to galleries/museums | -0.02 | **0.45** | 0.20 | 0.06 | 0.19 | 0.17 | 0.01 | -0.17 |
| Attend public talk | 0.01 | **0.56** | 0.20 | 0.19 | 0.21 | 0.06 | 0.04 | -0.03 |
| Attend sports events | 0.23 | 0.10 | 0.15 | 0.01 | 0.12 | 0.01 | -0.02 | **0.53** |
| Talk on phone to friends/relatives | -0.14 | 0.03 | 0.13 | 0.15 | 0.03 | **0.37** | 0.08 | 0.03 |
| Visit relatives/friends | -0.08 | 0.07 | 0.13 | 0.04 | 0.01 | **1.00** | 0.09 | -0.04 |
| Go out with friends | -0.17 | 0.07 | 0.05 | 0.03 | -0.05 | **0.36** | 0.11 | -0.07 |
| Attend religious services | -0.03 | 0.13 | 0.05 | **0.91** | -0.03 | 0.02 | 0.02 | 0.01 |
| Engage in prayer/meditation | 0.02 | 0.21 | 0.13 | **0.56** | 0.14 | 0.04 | -0.12 | -0.15 |
| Attend organised social events | -0.04 | 0.22 | 0.11 | **0.49** | 0.09 | 0.17 | -0.01 | -0.05 |
| Engage in political activities | 0.18 | **0.47** | 0.10 | 0.06 | 0.17 | 0.02 | -0.06 | 0.02 |
| Give public talk | 0.14 | **0.52** | 0.01 | 0.13 | 0.14 | -0.05 | 0.10 | 0.21 |
| Travel outside region | 0.07 | 0.04 | 0.12 | 0.02 | 0.09 | 0.08 | **0.72** | -0.01 |
| Travel outside town | 0.11 | 0.04 | 0.08 | -0.02 | 0.14 | 0.07 | **0.61** | 0.04 |

*Note*. **Bold** = loading > .3. Abbreviated versions of the VLS-ALQ items are included with permission to support the analyses; access to the VLS-ALQ and permission to use the scale in full or in part must be obtained from Professor Roger Dixon (rdixon@ualberta.ca).
