# Supplementary material for: Measuring activity engagement in old age: An exploratory factor analysis
Source: PLoS One. 2021 Dec 6;16(12):e0260996. doi: 10.1371/journal.pone.0260996 (PMC8648112; doi:10.1371/journal.pone.0260996)
Supplement: S7 Appendix — (DOCX) [file pone.0260996.s007.docx]

**S7 Appendix**

**Complete Case Analysis: Six-Factor Model Pattern/Structure Matrices**

**Table A. Six-Factor Model Pattern Matrix**

|  | Factor | | | | | |  |
| --- | --- | --- | --- | --- | --- | --- | --- |
| Item | 1. Man | 2. Int | 3. Gam | 4. Rel | 5. Exe | 6. Soc | *h^2^* |
| Do household repairs (for example, painting or leaky faucets) | **0.91** | -0.04 | 0.02 | 0.01 | 0.03 | 0.02 | 0.82 |
| Repair a mechanical device (for example, a car or lawn mower) | **0.74** | 0.07 | -0.06 | -0.02 | -0.03 | 0.03 | 0.57 |
| Purchase a new item requiring some set-up or assembly | **0.68** | 0.03 | 0.10 | 0.01 | 0.05 | -0.07 | 0.50 |
| Engage in creative writing, writing poems, writing newspaper articles, etc. | 0.16 | **0.55** | -0.08 | 0.05 | -0.10 | 0.04 | 0.34 |
| Read books or magazines as part of my job, career, or formal education | 0.07 | **0.51** | 0.04 | -0.09 | -0.02 | 0.04 | 0.28 |
| Go to galleries or museums | -0.11 | **0.52** | 0.10 | -0.01 | 0.10 | 0.04 | 0.33 |
| Attend a public lecture or talk | -0.09 | **0.63** | 0.08 | 0.06 | 0.09 | -0.03 | 0.46 |
| Engage in political activities (for example, neighbourhood organisation) | 0.07 | **0.51** | -0.05 | -0.08 | -0.03 | -0.01 | 0.26 |
| Give a public talk or lecture (for example, to a club, service organisation, etc.) | 0.08 | **0.47** | -0.12 | 0.09 | -0.08 | -0.03 | 0.24 |
| Do aerobics (for example, cardiovascular, fitness training, or workout) | 0.07 | -0.02 | 0.01 | 0.05 | **0.63** | -0.01 | 0.41 |
| Do flexibility training (for example, stretching, yoga, or tai chi) | -0.09 | 0.09 | 0.07 | 0.02 | **0.53** | 0.03 | 0.32 |
| Do weight lifting, strength training, or calisthenics | 0.04 | -0.02 | -0.07 | -0.05 | **0.76** | 0.00 | 0.59 |
| Play card games (for example, Bridge) | -0.03 | -0.03 | **0.47** | 0.06 | 0.10 | 0.02 | 0.24 |
| Play board games (for example, chess or checkers) | 0.08 | 0.08 | **0.63** | -0.05 | -0.01 | -0.09 | 0.41 |
| Play knowledge games (for example, Trivial Pursuit) | 0.03 | 0.04 | **0.66** | 0.00 | -0.05 | 0.00 | 0.45 |
| Play word games (for example, Scrabble) | -0.01 | -0.07 | **0.71** | 0.01 | -0.03 | 0.07 | 0.51 |
| Talk on the phone to friends, or relatives | -0.09 | -0.01 | 0.10 | 0.14 | 0.04 | **0.40** | 0.23 |
| Visit relatives, friends, or neighbours | 0.02 | 0.00 | 0.00 | -0.01 | 0.00 | **1.00** | 1.00 |
| Go out with friends | -0.17 | 0.15 | 0.05 | 0.00 | 0.03 | **0.37** | 0.23 |
| Attend church or other religious services | 0.02 | -0.03 | -0.01 | **0.97** | -0.03 | -0.01 | 0.93 |
| Engage in prayer, meditation, or philosophical contemplation | -0.04 | 0.10 | 0.05 | **0.55** | 0.11 | 0.00 | 0.36 |
| Attend organised social events (for example, activities at the community centre or church social groups) | -0.06 | 0.17 | 0.00 | **0.47** | 0.04 | 0.10 | 0.30 |

*Note*. Man = Manual, Int = Intellectual, Gam = Games, Rel = Religious, Exe = Exercise, Soc = Social; **Bold** = loading > .3; *h^2^* = communality. VLS-ALQ items are included with permission to support the analyses; permission to use the VLS-ALQ in full or in part must be obtained from Professor Roger Dixon (rdixon@ualberta.ca).

**Table B. Six-Factor Model Structure Matrix**

|  | Factor | | | | | |
| --- | --- | --- | --- | --- | --- | --- |
| Item | 1. Man | 2. Int | 3. Gam | 4. Rel | 5. Exe | 6. Soc |
| Do household repairs (for example, painting or leaky faucets) | **0.90** | 0.09 | 0.03 | -0.05 | 0.12 | -0.09 |
| Repair a mechanical device (for example, a car or lawn mower) | **0.75** | 0.15 | -0.04 | -0.05 | 0.05 | -0.06 |
| Purchase a new item requiring some set-up or assembly | **0.70** | 0.14 | 0.10 | -0.03 | 0.13 | -0.12 |
| Engage in creative writing, writing poems, writing newspaper articles, etc. | 0.21 | **0.55** | 0.02 | 0.13 | 0.01 | 0.08 |
| Read books or magazines as part of my job, career, or formal education | 0.13 | **0.51** | 0.12 | -0.01 | 0.09 | 0.11 |
| Go to galleries or museums | -0.03 | **0.55** | 0.19 | 0.08 | 0.19 | 0.14 |
| Attend a public lecture or talk | 0.00 | **0.66** | 0.19 | 0.17 | 0.19 | 0.09 |
| Engage in political activities (for example, neighbourhood organisation) | 0.14 | **0.49** | 0.02 | 0.00 | 0.06 | 0.04 |
| Give a public talk or lecture (for example, to a club, service organisation, etc.) | 0.13 | **0.46** | -0.05 | 0.15 | 0.00 | 0.01 |
| Do aerobics (for example, cardiovascular, fitness training, or workout) | 0.13 | 0.11 | 0.04 | 0.03 | **0.64** | 0.02 |
| Do flexibility training (for example, stretching, yoga, or tai chi) | -0.03 | 0.19 | 0.12 | 0.03 | **0.54** | 0.09 |
| Do weight lifting, strength training, or calisthenics | 0.12 | 0.10 | -0.03 | -0.08 | **0.76** | 0.02 |
| Play card games (for example, Bridge) | -0.02 | 0.07 | **0.48** | 0.08 | 0.11 | 0.10 |
| Play board games (for example, chess or checkers) | 0.10 | 0.17 | **0.62** | -0.01 | 0.04 | 0.01 |
| Play knowledge games (for example, Trivial Pursuit) | 0.04 | 0.14 | **0.67** | 0.04 | 0.00 | 0.10 |
| Play word games (for example, Scrabble) | -0.03 | 0.05 | **0.71** | 0.04 | 0.00 | 0.17 |
| Talk on the phone to friends, or relatives | -0.14 | 0.08 | 0.17 | 0.17 | 0.05 | **0.44** |
| Visit relatives, friends, or neighbours | -0.10 | 0.13 | 0.15 | 0.05 | 0.05 | **1.00** |
| Go out with friends | -0.19 | 0.19 | 0.13 | 0.06 | 0.06 | **0.42** |
| Attend church or other religious services | -0.05 | 0.12 | 0.03 | **0.96** | -0.06 | 0.04 |
| Engage in prayer, meditation, or philosophical contemplation | -0.05 | 0.22 | 0.10 | **0.57** | 0.11 | 0.06 |
| Attend organised social events (for example, activities at the community centre or church social groups) | -0.07 | 0.26 | 0.07 | **0.51** | 0.06 | 0.16 |

*Note*. Man = Manual, Int = Intellectual, Gam = Games, Rel = Religious, Exe = Exercise, Soc = Social. **Bold** = loading > .3. VLS-ALQ items are included with permission to support the analyses; permission to use the VLS-ALQ in full or in part must be obtained from Professor Roger Dixon ([rdixon@ualberta.ca](mailto:rdixon@ualberta.ca)).
