# Supplementary material for: Measuring activity engagement in old age: An exploratory factor analysis
Source: PLoS One. 2021 Dec 6;16(12):e0260996. doi: 10.1371/journal.pone.0260996 (PMC8648112; doi:10.1371/journal.pone.0260996)
Supplement: Attachment — Submitted filename: Response to Reviewers 01.10.2021.docx [file pone.0260996.s008.docx]

Editor’s comments to author:

Thank you for submitting your manuscript to PLOS ONE. After careful consideration, we feel that it has merit but does not fully meet PLOS ONE’s publication criteria as it currently stands. Therefore, we invite you to submit a revised version of the manuscript that addresses the points raised during the review process.

…

We look forward to receiving your revised manuscript.

Kind regards,

Mohammad Asghari Jafarabadi

Academic Editor

PLOS ONE

**RESPONSE:** We would like to express our appreciation to both reviewers for providing helpful and constructive comments. We agree with many of the comments and have made revisions (outlined below) that we hope sufficiently address the concerns raised. Where we felt that we could not make changes that reviewers have requested, we have also explained this below.

**Note:** We have included one additional reference in the revised manuscript (reference no. 35). Line numbers included below refer to the revised manuscript with tracked changes.

Reviewer #1: The reviewed paper discusses inconsistency in the definition of activity engagement domains in studies of cognitive health in advanced age. To this end, the authors performed an exploratory factor analysis on data collected from adults age 65+ in the UK using an adapted version of the VLS-ALQ.

I commend the authors for their effective communication of the rationale, methods, and results of their work. The methods were particularly well-described and justified, which is important given the authors encourage testing these study findings in different populations. While this research alone cannot create census on what domains are most appropriate (which the authors themselves point out), it does lay a good methodological foundation for how those domains should be defined. This foundation could, in turn, lead to greater consistency when classifying domains for studies on cognitive health in advanced age.

There are several strong limitations of this study, but I believe they are adequately covered by the authors in the discussion section.

**RESPONSE:** We thank the reviewer for their comments. We particularly appreciate the comment that though there are limitations to the study, we have acknowledged those. We are in full agreement too with their sense of the purpose of our paper: not to present a final resolution of the issue but rather to raise that in a data-driven way to support further careful consideration, and we hope in time, consensus on the best measurement of activity and engagement. We hope, as noted by the reviewer, that these findings will provide a foundation for future research.

At line [174] I would like to know if the research team members received standardized training before administering the assessments.

**RESPONSE:** We agree that it is important for the reader to know the degree of standardisation in the assessments conducted. All members of the research team were trained in the administration of all cognitive and physical tests prior to starting data collection. We have added this information to the manuscript in lines 177-178, which reads:

*“All members of the research team were trained in the administration of these standardised tests prior to the commencement of data collection.”*

Within the limits of my expertise, I think this is solid work and should be accepted for publication.

**RESPONSE:** We are grateful for the support for our manuscript, and hope the changes made help to add some useful additional details.

Reviewer #2: Review of the manuscript titled: Measuring activity engagement in old age: an exploratory factor analysis

Dear Editor

Thank you for allowing me to review the manuscript for this Research Article

1. The language, flow and the content of this paper are well written.

**RESPONSE:** We thank the reviewer for their positive comment, and hope that the revisions below address their remaining concerns.

2. Materials and Methods line 117 to 119 not captured in Table 1. I recommend this information to be added in the same table.

**RESPONSE:** The eighty participants described in these lines who were not included past the screening phase did not consent to take part in the full study, and thus did not provide the data summarised in Table 1. Given the data are not available for non-participants, unfearingly the table cannot be updated to include these individuals. In order to make clear that these individuals are not considered part of the analytic sample, we have revised this section slightly. The section now reads as follows (lines 117-120):

*“A total of 416 individuals were screened for eligibility. Eighty did not meet the criteria and were excluded or withdrew from the study after screening. The remaining 336 volunteers were enrolled on the study and comprise the analytic sample (102 males, 234 females; for sample characteristics see Table 1).”*

3. In statistical analysis part, I recommend to show how you addressed the multicollinearity of different domains/items.

**RESPONSE:** We agree that it is important to acknowledge the issue of multicollinearity. In order to examine multicollinearity among the items included in the analysis, we calculated the determinant of the correlation matrix (as recommended by Yong and Pearce, reference 35 in the revised manuscript). A value of greater than .00001 indicates that there is no problematic multicollinearity among the items. We have added this detail to the Statistical Analysis section on lines 216-219, along with a new reference to the Yong and Pearce paper. We also took this opportunity to add the reasoning behind carrying out Bartlett’s test in the same section. The revised section now reads (lines 216-219):

*“Bartlett’s test was conducted to ensure that correlations between these items were of sufficient size for EFA. The determinant of the item correlation matrix was calculated to assess multicollinearity among the items (a value > .00001 can be taken as indicating an absence of multicollinearity) [35].”*

We observed that the value of the determinant of the correlation matrix in this study was .002, greater than the cut-off recommended by Yong and Pearce. This indicates that there was no excessive multicollinearity among the items included in the EFA. We have acknowledged this in lines 246-247, which now reads:

*“The determinant of the correlation matrix did not indicate any problem with multicollinearity among the items (determinant = .002).”*

Regarding any possible multicollinearity among the factors, we note that the factor correlations presented in Table 3 are small (the largest is between the Intellectual and Exercise domains, *r* = .26). Overall, there is therefore little evidence of multicollinearity. We have added a short reference to this on lines 293-294-301, which now reads:

*“Correlations between the factors were generally small, ranging in magnitude from -.10 to .26 (see Table 3).”*

4. In the result section: Line 233- As an exploration factor analysis it is important to show the distribution figure (to represent different pattern; normal and non-normal, skewness, kurtosis)

**RESPONSE:** We appreciate the reviewer’s comment and agree that the distribution of the items is important. As highlighted in the Statistical Analysis section, the multiple imputation generated five imputed datasets with varying estimates for missing values. This means that each individual item had different distributions in each dataset. As noted in lines 210-212, skew and kurtosis values for each item in each imputed dataset were examined, and items were excluded if they had an absolute skew value greater than 2 across all imputed datasets and/or an absolute kurtosis value greater than 7 across all imputed datasets (these values typically indicate a significant deviation from normality). As a result of this, eight items were excluded. Presenting the distributions of each of these items in each imputed dataset would require presenting 40 different distributions (8 items across 5 imputations).

Rather than include such a large number of figures, we have added a note on lines 238-239 to re-emphasise the criteria for excluding items for non-normal distributions. This now reads:

*“Eight items were initially excluded due to highly non-normal distributions (i.e., absolute univariate skew > 2 and/or absolute univariate kurtosis > 7, detailed above).”*

We have also amended S2 Appendix to include the range of skew and kurtosis values across imputed datasets for the excluded items, which we hope provides the relevant information requested. We hope that these changes help the reader to better understand how significant the deviation from normality was for these items.

5. Discussion: Line 371-372 include this (r = .26, p < .001) to support your discussion that there was a significant positive correlation between scores in the Exercise domain and total MET-minutes per week from the IPAQ.

**RESPONSE:** As suggested by the reviewer, we have added the corresponding r and p values to the relevant section of the Discussion (now line 366). We have also taken the opportunity to further discuss a possible reason why the observed correlation, while significant and in the expected direction, was small in size. The revised section on lines 368-372 now reads:

*“Some evidence of construct validity was found. Of particular note was the significant positive correlation (r = .26, p < .001) between scores in the Exercise domain and total MET-minutes per week from the IPAQ (a broader measure of overall physical activity). As noted above, the Exercise domain of the VLS-ALQ measures frequency of engagement in specific fitness-related activities, while the measure of MET-minutes per week from the IPAQ considers the intensity of physical exertion across a wider range of activities. This may explain that while the observed correlation was in the expected direction, it was small in magnitude.”*

6. Finally, I’m pretty convinced to include as an appendix or a link for the Victoria Longitudinal Study-Activity Lifestyle Questionnaire (VLS-ALQ) in this manuscript.

**RESPONSE:** We appreciate the reviewer’s comment, and while we would be happy to include the full questionnaire, we do not have permission to do so. Access to the VLS-ALQ is managed by the Victoria Longitudinal Study team. We have noted this in both the notes for Table 3 and the acknowledgement section, which also provides necessary contact details for those wishing to access the questionnaire.
